# Violence –related injuries in a rapidly developing Middle Eastern country: a retrospective study from a level 1 trauma center

**DOI:** 10.1186/s12889-020-09754-7

**Published:** 2020-11-03

**Authors:** Monira Mollazehi, Ayman El-Menyar, Ahammed Mekkodathil, Rafael Consunji, Hassan Al-Thani

**Affiliations:** 1 Department of Surgery, Trauma Surgery, Hamad General Hospital, Doha, Qatar; 2 Department of Clinical Medicine, Weill Cornell Medical College, Doha, Qatar; 3 Trauma & Vascular Surgery Section, Hamad General Hospital, Hamad Medical Corporation & Weill Cornell Medical College, PO Box 3050, Doha, Qatar

**Keywords:** Interpersonal violence, Self-inflicted violence, Trauma, Injury, Qatar

## Abstract

**Background:**

Violence is a global public health concern leading to injuries, long-term physical, sexual or mental health problems and even mortality. The burden of violence-related injuries on hospital systems remains understudied in the Arabian Gulf region. The present study aimed to describe the epidemiology of hospitalized violence-related injuries in a rapidly developing Middle Eastern country.

**Methods:**

A retrospective analysis from a level 1 trauma center, in the state of Qatar, was conducted. Data were retrieved from the Qatar national trauma registry for all patients who were admitted with violence-related injuries between June 2010 and June 2017. Analyzed data were used to compare hospitalized interpersonal and self-inflicted violence groups.

**Results:**

The hospitalization rate of violence-related injuries was 4.6 per 100,000 population per year; it was significantly higher in males (5.5/100,000 males/year vs. 1.8/100,000 females/year) and younger persons, particularly in the 25–34 years old population (41%). South Asians constituted 55% of the affected study population. Interpersonal violence (76.7%) was the most common mechanism of injury. Significant differences between interpersonal and self-inflicted violence groups were evident, especially for the type of trauma (i.e. blunt or penetrating), injured body regions, alcohol use, injury severity, need for intubation and psychiatric referral (*p* < 0.05). Overall, in-hospital mortality was 6.4%; with a significantly higher rate in females (16% vs.5%, *p* = 0.001). Outcomes, including length of hospital stay and mortality, were comparable between the two study groups. Multivariate analysis showed that male gender and alcohol use were predictors for interpersonal violence whereas high Injury Severity Score (ISS) and low Glasgow Coma Scale (GCS) were predictors of hospital mortality.

**Conclusions:**

The rate of hospitalization for violence-related injuries in Qatar is low; however, its burden on the trauma system is of concern. Although it comprised only 9.6% of the study population, females are more likely to get hospitalized following self-inflicted injuries when compared to interpersonal violence. The disproportionate burden of violence among South Asian and young populations warrants an evidence-based public health approach to appropriately address the risk factors and set prevention programs.

**Supplementary Information:**

The online version contains supplementary material available at 10.1186/s12889-020-09754-7.

## Introduction

Violence-related trauma refers to injuries resulting from an intentional use of physical force or power against oneself or others. The World Health Organization (WHO) defined violence broadly by incorporating self-directed violence; interpersonal violence (IPV) and collective violence leading to injury or with a high probability of contributing to injury, death, psychological harm, maldevelopment or deprivation [1]. In 2000, it was estimated that over 1.6 million deaths (28.8 per 100,000 population) worldwide were attributed to violence, with many more injured and/or suffering from long-term physical, sexual or mental health disability or conditions [1].

Worldwide data showed that young (15–44 years) and male gender (14% vs. 7% for females) made up the majority of violence-related mortality [2]. Interpersonal violence remains as a major contributor (30.5%) to violence-related mortality across the globe with a rate of 8.8 per 100,000 population [1]. Nearly, 1.2 million deaths in 2013 were related to intentional injuries in which IPV constituted 32% whereas self-directed violence accounted for 68% [3]. Interpersonal violence includes family and intimate partner violence (child, partner or elder) and community violence (stranger or acquaintance). The lifetime prevalence of intimate partner violence in women in the United States was estimated at 28 to 54% [4, 5]. The prevalence of sexual abuse in children was reported as 3 to 40% based on reports from Canada and Switzerland [6, 7].

Each type of violence might have different set of determinants and contributory factors; however, there are some common factors across the types of violence. Certain demographics, use of drugs and alcohol, access to firearms, gender, and social or economic inequalities in the distribution of or use of power, increase the risk for violence [8]. In addition to mortality from violence, the morbidity and associated economic costs require focused preventive public health approaches based on interdisciplinary consensus and scientific evidence [2].

It was demonstrated that approximately 20–40 victims of non-fatal youth violence receive hospital treatment for every youth homicide [9]. The economic cost associated with violence was estimated as nearly 3.3% of the gross domestic product (GDP) in the United States [10]. Intimate partner violence accounted for 1.6% of the GDP lost in Nicaragua and 2% of GDP lost in Chile [10]. In England and Wales, the cost of violence was estimated as approximately £20 billion per year [10]. In the US, the direct medical cost associated with child abuse alone, was between 13,781 and 42,518 USD per abused child [10].

The healthcare burden of violence-related injuries in the western countries is well documented, but remains understudied in the Middle Eastern region, especially in the Arabian Gulf countries. Published studies on violence-related injuries especially those requiring hospitalization in Qatar are not available. The government of Qatar has developed several strategies to increase the efficiency in reporting violence, especially domestic violence. Community awareness programs were identified as crucial to provide a safe environment to report such incidents.

The healthcare system of Qatar provides high levels of healthcare in advanced medical facilities for all, regardless of residency, nationality or socio-economic status. Charges for medical services are waived for all trauma patients entering the health system via emergency services by an Amiri Decree that mandates care provided for all trauma patients to be free of charge throughout their hospitalization.

There is a great need to address the existing gaps in violence-related injuries documentation in the country to empower stakeholders and inform policy makers. This will contribute to the development of evidence-based strategies and ultimately strengthen the capacity of Qatar’s health system to prevent violence-related injury and its consequences. This study aims to describe the epidemiology and pattern of violence-related injuries that required hospitalization at Hamad Trauma Center (HTC), the only tertiary level 1 trauma center in the state of Qatar.

## Methods

A retrospective analysis was conducted for data obtained from the Qatar National Trauma Registry (QTR) at HTC. QTR is a mature database that participates in both the National Trauma Data Bank and Trauma Quality Improvement Program of Committee on Trauma of the American College of Surgeons (TQIP-ACS). The study received ethical approval from the Institutional Review Board (IRB) of Hamad Medical Corporation (#MRC-01-18-189). The HTC is the only level 1 trauma center in Qatar, which sees and treats moderate to severely injured patients across the country including referrals from other hospitals. Each year, a trauma code (Level I, II or III Trauma Criteria) is activated for nearly 2500 patients. Of these patients, an average of 1500–1800 require hospital admission at the HTC, 500–700 patients receive treatment at the ED without need for hospitalization and an average of 80 patients die before arrival because of their injuries. This study included all patients admitted to the level 1 HTC for violence related (IPV and self-inflicted) injuries from 1 June 2010 to 30 June 2017. Patients who were brought dead, died on arrival to the hospital or did not require hospital admission were excluded from the final analysis. Patients were categorized into two groups based on the type of violence (interpersonal and self-inflicted) and their demographics, injury characteristics, management and in-hospital outcomes were analyzed and compared.

Extracted data included age, gender, nationality, mechanism of injury, Glasgow Coma Scale (GCS), Ethanol level (blood alcohol concentration), injured regions, injury severity score (ISS), major procedures and outcome. The Glasgow Coma Scale (GCS) is a neurological scale with scores that range from 3 to 15 to assess consciousness in which GCS ≤ 8 severe, 9–12 moderate and ≥ 13 minor head injuries [11]. The term “alcohol” denotes “ethyl alcohol or ethanol”. Blood alcohol concentration (BAC) was reported as millimoles of ethanol per liter of blood (mmol/L). Any BAC level above zero mmol/L was reported as BAC-positive; levels 0.1–10.9 were “less intoxicated”; 10.9–21.7 were “intoxicated”, and > 21.7 were “very intoxicated” (or at CNS depression levels) [12].

The Abbreviated Injury Scale (AIS) refers to the severity of injuries in different body regions; scores range from 1 to 6, representing minor, moderate, serious, severe, critical and non-survivable injuries respectively [13, 14]. The AIS scores of the 3 most severely injured body regions are squared and added together to estimate the Injury Severity Score (ISS) in order to provide an overall score for polytrauma [13, 14]. ISS scores range from zero to 75 where 0–9 is minor; 10–15 moderate; 16–24 severe; and > 25 is critical [13]. Data elements are abstracted concurrently. Injury details are obtained from the final radiology reports, operative notes as well as physician and nursing documentations. The abstracted injury data are used for the AIS and ISS calculations during the patient’s hospitalization.

Data for population in Qatar were obtained from the website of the Ministry of Development, Planning and Statistics in Qatar [15], from which the mid-year population was used for estimating the average annual incidence rate of violence related hospital admission.

Patients data were identified from the QTR through the electronic medical records, reviews patient history, searches for police documentation, social worker notes and referrals to the Women and Child Protection Team for the confirmation of a violent event. The trauma registry utilizes the Classification of External Cause of Injury and Poisoning (E-Codes) of the International Classification of Diseases, Ninth Revision, Clinical Modification (ICD-9-CM) in the study period. The E-codes for suicide and self-inflicted injury (E950-E959) included: injuries in suicide and attempted suicide, self-inflicted injuries specified as intentional. The codes for homicide and injury purposely inflicted by other persons (E960-E969) included: injuries inflicted by another person with intent to injure or kill, by any means. The codes for legal intervention (E970-E978) included: injuries inflicted by police or other law enforcing agents**.** Each patient record was given a unique study number, and patient anonymity was maintained throughout the study. The trauma registry data have internal and external validation on regular basis. This manuscript adheres to the Strengthening the Reporting of Observational studies in Epidemiology (STROBE) guidelines (Suppl table) [16].

### Statistical analysis

Descriptive and bivariate analysis of trauma registry data were carried out based on the inclusion and exclusion criteria in the study period. Data were summarized in form of proportions for categorical variables and, mean (± standard deviation) and median (range) for continuous variables. Categorical variables were compared using Chi-square test and Fisher exact test depending on the size of the data set. Independent student t-test was used for continuous variables. Multivariate analysis models for predictors of the type of violence and predictors of mortality were performed using the relevant and significant variables such as age, gender, nationality, mechanism and type of injury, injury severity score, admission GCS, and alcohol consumption (BAC status) and data were expressed as odds ratios (OR) and 95% confidence intervals (CI). The Hosmer and Lemeshow test was used for *goodness of fit* for logistic regression models. A two-tailed *P* value of < 0.05 was statistically significant. All data analyses were carried out using the Statistical Package for the Social Sciences, version 18 (SPSS, Inc., Chicago, IL).

## Results

**Fig. 1:**
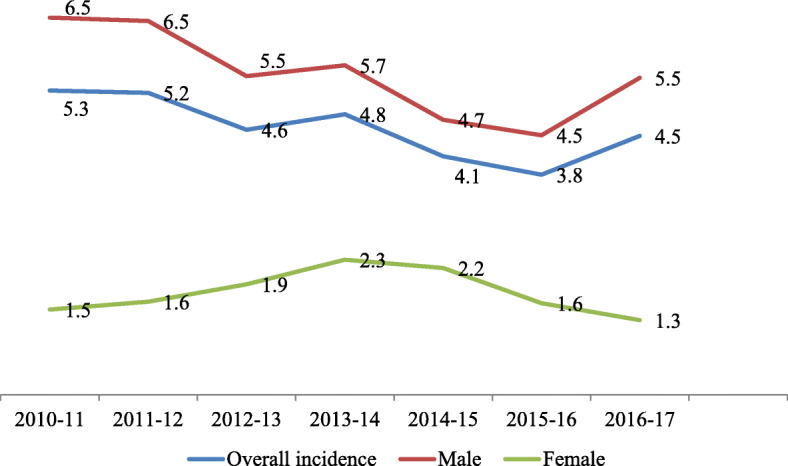
Hospitalization rate for violence-related traumatic injuries by gender in Qatar (2010–2017)

The study identified 658 hospitalized patients (595 (90.4%) males & 63 (9.6%) females) who sustained violence-related injuries in the study period. The hospital admission rate, for violence-related traumatic injuries, was 4.6 per 100,000 population per year. This rate was higher among males (5.5 per 100,000 males per year) when compared to females (1.8 per 100,000 females per year). Females represented less than 10% of the hospitalized violence related victims. The total population of Qatar increased nearly 60% across the study duration, but the population growth rate was comparable in both genders. Although the number of injured patients increased from 86 to 116 in 2010 and 2017 respectively, the corresponding hospitalization rate showed a 15% decrease from 5.3 to 4.5 per 100,000 population (Table 1 & Fig. 1).

**Table 1:** Number and rate of hospitalization for violence-related injuries by gender in Qatar (2010–2017)

	Number of admissions at the trauma center			General population in Qatar (Mid-year)		
Duration	Males	Females	Total	Males	Females	Overall
2010–2011	80	6	86	1,228,635	408,808	1,637,443
2011–2012	82	7	89	1,271,194	436,562	1,707,756
2012–2013	75	9	84	1,364,063	472,613	1,836,676
2013–2014	87	12	99	1,530,101	515,138	2,045,239
2014–2015	80	12	92	1,686,228	549,203	2,235,431
2015–2016	83	9	92	2,423,175	1,853,001	570,174
2016–2017	108	8	116	2,597,453	1,974,699	622,754
Total number/ Rate of hospitalization	595	63	658	4.6	5.5	1.8

**Fig. 2:**
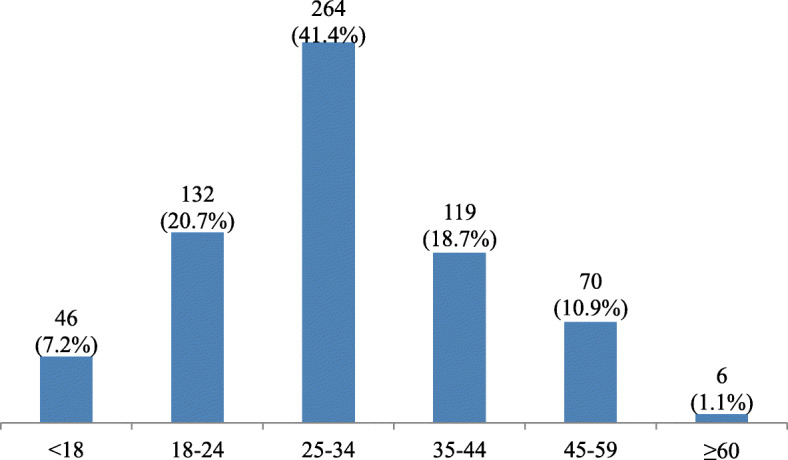
Number (percentage) of violence-related injuries by age-group of patients admitted at the Hamad Trauma Center, Doha, Qatar (2010–2017)

The mean age of patients was 31 years ranging from < 2 years to 77 years. Approximately 7% were children; under 18 years. Most of the affected persons were in the age group between 25 and 34 years old (41%). The elderly population, aged over 60 years, represented around 1% of the study patients (Fig. 2).

The type of violence was not documented in 48 cases and most of injuries were interpersonal violence-related (76.7%) followed by self-inflicted injuries (23.3%). Although males were predominant in any type of violence; the proportion of males involved in IPV were significantly higher when compared to self-inflicted injuries [SII] (*p* = 0.001) (Table 2). Females were more commonly exposed to self-inflicted violence compared to the interpersonal type. More than half (55%) of IPV-related injuries were reported in patients from South Asia. Nearly 11% were reported among Qatari nationals. The type of trauma was almost equally distributed between blunt and penetrating injuries. However, there was a significant difference by type of violence; penetrating trauma was more common in IPV whereas blunt trauma was more frequent in SII (*p* = 0.003).

**Table 2:** Characteristics and outcomes of patients admitted to the trauma center following violence-related injuries

	Overall^a^	Interpersonal (76.7%)	Self-inflicted (23.3%)	*P*-value
Age; mean ± SD	30.8 ± 11.1	30.7 ± 11.5	30.4 ± 8.9	0.004
Males; n (%)	595 (90.4)	446 (95.3)	102 (71.8)	0.001
Type of trauma; n (%)				
• Blunt	331 (50.5)	229 (48.9)	76 (53.5)	0.003
• Penetrating	324 (49.5)	239 (51.1)	63 (44.4)	
GCS; mean ± SD				
• ED	13.63 ± 3.0	13.45 ± 3.8	13.29 ± 4.10	0.001
• After 1 h	13.29 ± 3.9	12.4 ± 4.67	11.96 ± 5.06	0.001
BAC Positive; n (%)	143 (23.4)	116 (24.8)	17 (12.0)	0.001
BAC; mean ± SD	36.9 ± 17.0	36.3 ± 16.9	37.8 ± 16.8	0.75
Injury at body sites; n (%)				
• Head	177 (26.9)	141 (30.1)	21 (14.8)	0.001
• Face	86 (13.1)	60 (12.8)	11 (7.7)	0.063
• Neck	48 (7.3)	21 (4.5)	27 (19.0)	0.001
• Chest	137 (20.8)	108 (23.1)	22 (15.5)	0.053
• Abdomen	161 (24.5)	116 (24.8)	36 (25.4)	0.891
• Spine	53 (8.1)	27 (5.8)	26 (18.3)	0.001
• Arm	59 (9.0)	40 (8.5)	15 (10.6)	0.462
• Pelvis	39 (5.9)	19 (4.1)	21 (14.8)	0.001
• Leg	57 (8.7)	33 (7.1)	23 (16.2)	0.001
• External	433 (65.8)	321 (68.6)	84 (59.2)	0.037
AIS; mean ± SD				
• Head	3.4 ± 0.9	3.5 ± 1.1	4.3 ± 2.2	0.008
• Face	1.8 ± 0.4	1.8 ± 0.5	1.9 ± 0.6	0.371
• Neck	2.4 ± 1.2	2.6 ± 1.8	2.7 ± 1.8	0.799
• Chest	2.7 ± 0.9	3.0 ± 1.6	2.9 ± 1.6	0.847
• Abdomen	2.3 ± 0.8	2.7 ± 1.6	2.2 ± 0.7	0.050
• Spine	2.3 ± 0.9	2.4 ± 1.0	2.2 ± 0.7	0.498
• Arm	1.9 ± 0.6	1.9 ± 0.6	2.0 ± 0.5	0.564
• Pelvis	2.2 ± 0.7	2.4 ± 1.7	2.4 ± 0.7	0.864
• Leg	2.2 ± 0.5	2.4 ± 1.3	2.2 ± 0.4	0.571
• External	1.1 ± 0.5	1.2 ± 0.7	1.3 ± 0.9	0.208
ISS; median (range)	9 (1–75)	9 (1–75)	5 (1–75)	0.05
Management; n (%)				
• Intubation	158 (24.0)	106 (22.6)	48 (33.8)	0.007
• Exploratory Laparotomy	122 (18.5)	86 (18.4)	29 (20.4)	0.585
• Thoracotomy	12 (1.8)	10 (2.1)	0	0.079
• Chest tube insertion	82 (12.5)	66 (14.1)	12 (8.5)	0.077
• Craniotomy/Craniectomy	23 (3.5)	21 (4.5)	2 (1.4)	0.092
• ORIF surgery	39 (5.9)	24 (5.1)	11 (7.7)	0.240
• Psychiatric referrals; n (%)	133 (20.2)	25 (5.3)	101 (71.1)	0.001
• ICU LOS; median (range)	3 (1–142)	3 (1–123)	4 (1–142)	0.451
• Hospital LOS; median (range)	4 (1–142)	3 (1–1032)	4 (0–142)	0.520
Mortality	42 (6.4)	26 (5.6)	13 (9.2)	0.125

*AIS* abbreviated injury score, *BAC* blood alcohol concentration, *LOS* length of stay, *ORIF* open reduction and internal fixation, *ISS* injury severity score

^a^Type of violence was not documented in 48 cases (excluded)

Blood alcohol screening revealed that 23% had a blood alcohol concentration (BAC) above zero; with a mean BAC level in the ‘very intoxicated’ range, 37 ± 17 mmol/L. The victims of IPV were more likely to be BAC positive than SII (25% vs. 12%, *p* = 0.001).

The mean GCS upon arrival to the trauma resuscitation unit was 13.0. Head, abdomen, chest, face and neck injuries were reported in 27, 24.5, 21, 13 and 7.3%, respectively. Head injuries were more common in IPV whereas neck, spinal and pelvic injuries were more likely in SII (*p* < 0.001). The mean ISS was 10 ± 9, mean head AIS (3 ± 1), chest AIS (3 ± 1) and abdomen AIS (2 ± 1). Although the proportion of head injured patients was higher in the IPV group; the head AIS was significantly higher in SII (*p* = 0.008). There was no significant difference between the 2 violence groups in terms of AIS for other body regions. However, the ISS showed that SII were mild when compared to IPV victims (*p* = 0.05).

Twenty eight percent of patients required ICU admission. Endotracheal intubation was required in 24% of cases followed by exploratory laparotomy (19%) and chest tube insertion (13%). The only significant difference in the management between interpersonal and SII were reported for endotracheal intubation; self-inflicted injury patients were more likely to be intubated (34% vs. 23%, *p* = 0.007).

Twenty percent of patients were referred for psychiatric evaluation. Nearly 70% of patients with SII were referred for psychiatric evaluation whereas it was only 5% in the IPV group (*p* = 0.001). The median hospital length of stay was 4 days, while the ICU length of stay was 3 days. Both hospital and ICU length of stay were comparable across the groups based on the type of violence. Overall, in-hospital mortality was 6.4%; this was statistically comparable between the 2 study groups (Table 2).

Multivariable logistic regression analysis using relevant variables (age, gender, BAC positivity and nationality) showed that male gender (OR 7.5; 95% CI 4.144–13.828) and BAC positivity (OR 2.4; 95% CI 1.327–4.316) were predictors for IPV whereas South Asian nationality (OR 0.36; 95% CI 0.213–0.626) was a predictor of SII (Table 3). In another multivariable analysis model using age, gender, mechanism of injury, type of violence, BAC status, ISS and GCS on admission, the predictors of mortality were ISS (OR 1.14;95% CI 1.079–1.203) and GCS (OR 0.75; 95% CI 0.666–0.848) as shown in Table 4.

**Table 3:** Multivariable analysis for predictors of interpersonal violence among hospitalized trauma patients

Variable	*P* value	Odd ratio	95% confidence interval
**Age** (years)^a^	0.389	1.009	0.989–1.034
**Gender** (male)	0.001	7.570	4.144–13.828
**Nationality**			
Arabs (reference)		1	
South Asian	0.001	0.365	0.213–0.626
Africans	0.070	0.414	0.159–1.076
Westerns	0.640	0.705	0.160–3.106
**BAC** (positive)	0.004	2.393	1.327–4.316

Hosmer and Lemeshow test: Chi-Square 7.9, DF 8, *p* = 0.44

^a^ continuous variables, *BAC* blood alcohol concentration

**Table 4:** Multivariable analysis for predictors of mortality among hospitalized violence related trauma patients

Variable	*P* value	Odd ratio	95% confidence interval
**Age** (years)^a^	0.919	1.003	0.950–1.058
**Gender** (male vs female)	0.371	0.480	0.096–2.395
**Type of violence** (IPV vs self-inflicted)	0.157	3.397	0.625–18.476
**Mechanism of injury** (blunt vs penetrating)	0.191	2.349	0.654–8.440
**Injury Severity Score**^**a**^	0.001	1.139	1.079–1.203
**Admission Glasgow coma scale**^**a**^	0.001	0.751	0.666–0.848
**BAC** (positive vs negative)	0.194	0.341	0.067–1.717

Hosmer and Lemeshow test: Chi-Square 3.15, DF 8, *p* = 0.92

^a^ continuous variables, *BAC* blood alcohol concentration

## Discussion

The present study describes the epidemiology, pattern and outcomes of hospitalized violence-related injuries in Qatar. Although the number of victims increased over the years, the hospitalization rate decreased by 15%, throughout the study period. The computed hospitalization rate was 4.6 per 100,000 population per year. A significantly higher rate of violence was found among males and the 25–34 years old age group. Notably, children (<18y) represented 7% of the victims. The South Asians were proportionately affected according to their population distribution in the country. As such, any future interventions to prevent violence in the country must be culturally and linguistically appropriate for most affected populations and lessons must be taken from successful programs from their home countries as well. Multivariate analysis showed that male gender and BAC positivity were predictors for IPV whereas South Asian nationality was a negative predictor of SII. One fifth of cases were referred for psychiatric consultation; three-quarters of them were self-inflicted violence victims. The admission GCS and ISS were independent predictors of mortality in hospitalized violence related trauma patients in our analysis.

Interpersonal violence was the major contributor in most of victims followed by SII. More than one out of five patients were shown to be under the influence of alcohol and had a mean BAC level corresponding to the central nervous system depression level. Head and chest injuries were the most common severe injuries. The ISS data showed that polytrauma was not frequent in our study population. As per our knowledge, this is the first trauma registry-based study conducted on hospitalized violence-related trauma in Qatar. Such studies are rare in the Arabian Gulf region. A hospital-based retrospective study in Jordan demonstrated that violence (71%) was the most frequent cause of ED visits followed by road traffic injuries (23%) [17].

Bala et al., studied the prevalence of physical fighting and its associated factors among the adolescent population in Qatar [18]. This study was based on a student health survey in school to determine the prevalence and factors associated with being engaged in a physical fight. On the other hand, authors from Saudi Arabia reported risk factors and forms of intimate partner violence against Saudi women based on a survey among participants attending primary healthcare (PHC) outpatient clinics [19]. In another study, based on self-administrated questionnaire in PHC in Saudi Arabia, domestic violence against women was considerable with a passive response [20].

Notably, the majority of patients in our study were victims of IPV. Similarly, Osman et al., from Al-Ain city in the United Arab Emirates (UAE) conducted a trauma registry-based study that was specific for IPV [21]. The authors estimated that the interpersonal violence-related hospitalization rate in Al-Ain as 6.7 per 100,000 population, which was higher than the estimated rate in our study [21]. The mean age of victims in the UAE and our study was similar (30 and 31 years respectively). Male predominance among violence victims was evident in our study (90%), and that from Jordan (87%) and UAE (85%) [17, 21]. Our study demonstrated that females were more likely to sustain SII when compared to the IPV group in which males were more prevalent. The population structure in Qatar showed that females made up approximately a quarter of the total population, this could explain the gender pattern among the injured population.

The present study showed that the mortality among SII was higher than IPV, however, this difference was not statistically significant. The overall in-hospital mortality was 6.4% which could be related to the severity of injuries. Nearly 28% of our patients required ICU admission; whereas the UAE based study revealed that less than 3% were admitted to the ICU [21]. There was no mortality reported in the UAE study as the majority had mild injuries.

Blunt injuries were common in our study population, especially SII. This contrasted with studies from level 1 trauma centers in Western settings where penetrating injuries, especially gunshots, were more common [22]. In addition to the differences in geographical, cultural and religious backgrounds in the Middle Eastern settings, factors such as urbanization, crime rates, and legislation concerning firearm use could contribute to the existing variations in the rate and type of violence. Dijkink et al., demonstrated that the proportion of admitted patients with gunshot wounds was almost twice as high in level 1 trauma centers in the United States when compared to level 1 trauma centers in Netherlands, even though the geographical areas in both countries had comparable urbanization and violence crime rates [22].

### Strength and limitation

The major strength of our study is its regular internal and external validation since the data are obtained from the Qatar national trauma registry and the only tertiary, level 1 trauma center in the country. In the process of submitting our registry data to TQIP, the submission file goes through validation and will be checked. A submission frequency report will be provided for reference, and the file will be rejected if any major errors are found for correction. The TQIP reports are reviewed to pick up any outliers and correct any errors that might have been missed and then resubmit the changes. Therefore, our findings are validated and representative of violence-related hospitalizations in Qatar.

The main limitation of the study is the retrospective design. Also, selection bias cannot be ruled out as in some situations; the victims may not be willing to report the occurrence of violence and patients with minor injuries may not be admitted or attend the HTC. Although the frequency of hospital visits following violence-related injuries by nationality data was available, the annual nationality-specific population data were unavailable and therefore the disproportionate burden of injuries was not estimated based on the rate. In addition, other important socio-economic data were unavailable; however, the available data addressed the main objectives of the study. The present study excluded those who died at the site of injury or on arrival to the ED and therefore data represented those with comparatively less severe injuries. Changes in population growth rates over the years due to the influx of foreign workers, especially males, recruited for the major development projects in Qatar could explain the changes in the rates of violence related injuries across the study period [23]. Testing for non-prescription drugs are not routinely performed at the HTC, as such their role in violence-related injuries cannot yet be established.

## Conclusions

The rate of hospitalization for violence-related injuries in Qatar is relatively low with a decreasing trend. However, the higher rate of hospitalization among South Asians and younger residents must be addressed with proven and culturally appropriate preventive programs. Alcohol use is identified as a risk factor for IPV. Pediatric hospitalization and mortality associated with violence-related injuries needs commensurate attention. Females comprise 9.6% of the victims and were more likely to get hospitalized following SII when compared to interpersonal violence. The disproportionate burden of violence among South Asian and young populations warrants an evidence-based public health approach to appropriately address the risk factors and set prevention programs.

## Supplementary Information

**Additional file 1.** STROBE 2007 (v4) Statement—Checklist of items that should be included in reports of cross-sectional studies.

## Data Availability

All data were shown in the study analysis and tables. Further data need approval from the Qatar national trauma registry and medical research center in HMC (contact: mrcinfo@hamad.qa).

## Abbreviations

ISS: injury severity score
GCS: Glasgow Coma Scale
BAC: blood alcohol concentration
